# Inexpensive transparent nanoelectrode for crystalline silicon solar cells

**DOI:** 10.1186/s11671-016-1533-3

**Published:** 2016-06-29

**Authors:** Qiang Peng, Ke Pei, Bing Han, Ruopeng Li, Guofu Zhou, Jun-Ming Liu, Krzysztof Kempa, Jinwei Gao

**Affiliations:** Institute for Advanced Materials (IAM) and Laboratory of Quantum Engineering and Quantum Materials, South China Normal University, Guangzhou, 510006 People’s Republic of China; Department of Physics, Boston College, Chestnut Hill, 02467 MA USA; Electronic Paper Displays Institute, South China Normal University, Guangzhou, Guangdong 510006 People’s Republic of China; Laboratory of Solid State Microstructures, Nanjing University, Nanjing, 210093 People’s Republic of China; Laboratory of Nanoscale Energy Conversion Devices and Physics, Department of Mechanical Engineering, The University of Hong Kong, Pokfulam, Hong Kong

**Keywords:** Metallic nanowire networks, Metal-assisted chemical etching, Antireflection coating, Photovoltaics, Crystalline silicon solar cells

## Abstract

We report an easily manufacturable and inexpensive transparent conductive electrode for crystalline silicon (c-Si) solar cells. It is based on a silver nanoparticle network self-forming in the valleys between the pyramids of a textured solar cell surface, transformed into a nanowire network by sintering, and subsequently “buried” under the silicon surface by a metal-assisted chemical etching. We have successfully incorporated these steps into the conventional c-Si solar cell manufacturing process, from which we have eliminated the expensive screen printing and firing steps, typically used to make the macro-electrode of conducting silver fingers. The resulting, preliminary solar cell achieved power conversion efficiency only 14 % less than the conventionally processed c-Si control cell. We expect that a cell with an optimized processing will achieve at least efficiency of the conventional commercial cell, but at significantly reduced manufacturing cost.

**Electronic supplementary material:**

The online version of this article (doi:10.1186/s11671-016-1533-3) contains supplementary material, which is available to authorized users.

## Background

Transparent conductive electrodes (TCE) are important components of photovoltaic and photonic devices, such as displays, light sources, detectors, and solar cells [1–6]. Most common TCE used today are based on doped metal oxides, such as indium tin oxide (ITO) [7, 8]. However, the relatively high cost, limited optoelectronic performance, and mechanical brittleness exclude these from many applications, such as in flexible displays [9, 10]. Recently, research progress in nanomaterials has opened new directions for alternative TCE: based on carbon nanotubes (CNT) [11, 12], graphene [13, 14], metal nanowires [15, 16], and metal grids [17, 18]. As an example, metal nanowires have been shown to combine the optoelectronic advantages with the low-cost manufacturing, including the high-through put roll-to-roll processing [10, 19]. In planar configurations, and on transparent, low dielectric constant substrates, their optical [17] and/or photovoltaic [20] performance has been comparable, or better than that of the commonly used ITO. Importance of the dielectric environment in controlling optical response of metallic nanoarrays has been also demonstrated [21].

One of the most important areas where development of novel TCE has been critical is the solar photovoltaics (PV). Currently, while an excellent suppression of the reflection is achieved in crystalline silicon (c-Si) solar cells by the chemical surface texturing combined with the anti-reflection coating (ARC), a moderate conductivity of the overdoped underlying silicon requires a dense network of macroscopic metallic connecting bars/fingers [22, 23]. These, in turn, shadow the active surface of the cell, compromising the efficiency. To address this issue, in our previous work, we have developed a TCE based on metallic nanowires assembled directly in the micro valleys of a textured c-Si surface, like that employed in crystalline solar cells [24]. These nanowires were formed from silver nanoparticles deposited directly on the surface, and subsequently transformed into wires by microwave or furnace sintering. This nanoparticle nanonetwork (NNN) achieved an impressive optoelectronic performance, with reflectance of ~16 % without ARC and <5 % with ARC, as well as a good in-plane electrical conductivity of ~15 ohms/sq [24]. A logical next step has been since to test this new TCE on a functional solar cell, by replacing the conventional screen-printed TCE. Here, we provide such a demonstration, made possible by an addition of a critically important step in the NNN processing: a “burial” of the assembled NNN under the silicon surface by metal-assisted chemical etching [25, 26]. The resulting buried NNN (BNNN) not only significantly improves the contact of the network to the silicon but also reduces the TCE reflectance, due to the hidden/cloaked nature of the buried network. We have successfully incorporated these steps of BNNN processing into the conventional c-Si solar cell manufacturing process, just after the junction formation and before the nitride deposition and processing. All the other processes remained unchanged, except that the expensive screen-printed TCE processing and firing steps have been eliminated. The resulting solar cell, not yet optimized, achieved power conversion efficiency only 14 % less than the conventionally processed c-Si control cell, but its manufacturing cost has been significantly reduced.

## Methods

**Fig. 1:**
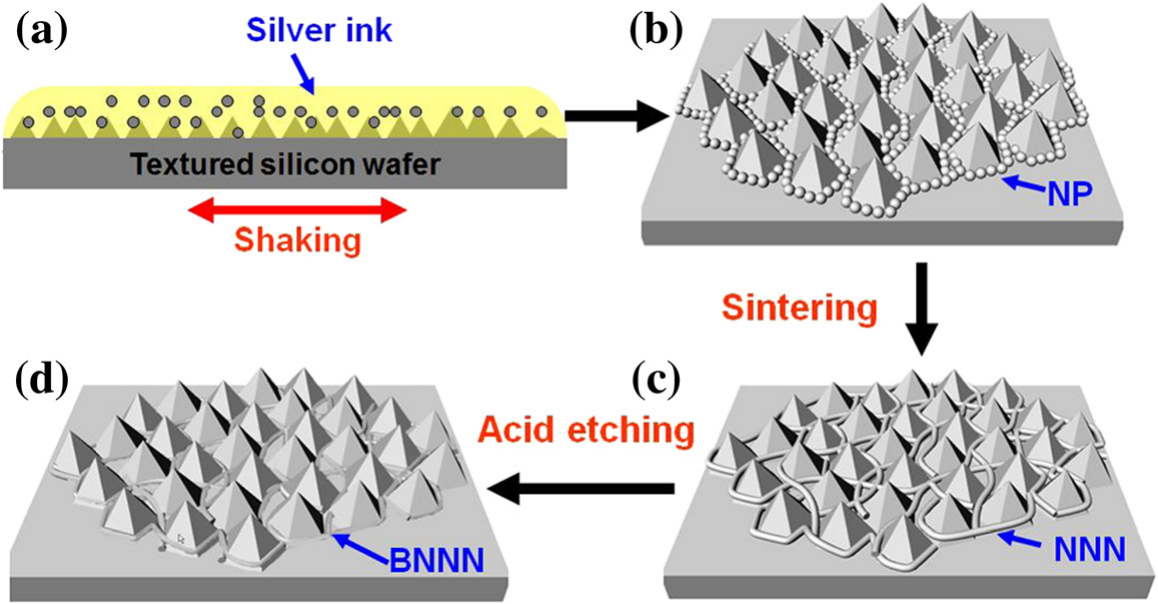
Schematic procedures for fabrication of BNNN TCE. **a** Deposition of silver ink film on the tSi surface. **b** Assembly/settling of NP in the valleys between the texture pyramids. **c** Formation of NNN by sintering. **d** Formation of BNNN by etching

The fabrication process of the BNNN includes four major steps: silver ink preparation, coating of the textured Si (tSi) surface with the ink film (Fig. 1a), assembly/settling of nanoparticles (NP) in the valleys between the texture pyramids (Fig. 1b), the sintering of NP to form NNN (Fig. 1c), and finally “burying” NNN by etching to form BNNN (Fig. 1d).

### Silver ink synthesis

The starting point in making BNNN TCE is the nanoparticle ink. The silver ink is produced by a typical wet-chemical method [27, 28], resulting in NP diameter of 100–200 nm. Making the ink involves reducing silver nitride (0.1 M) (99 %, Sigma-Aldrich) in an ethylene glycol solution in the presence of polyvinylpyrrolidone (PVP) (0.6 M) (MW ≈ 40000, Sigma-Aldrich) at 170 °C and stirring at 2000 rpm for 30 min. The ethylene glycol is both a reducer and a solvent, and PVP is a surfactant. This procedure is subsequently followed by centrifuging, rinsing, and re-dispersing the silver nanoparticles in methanol or ethanol.

### Wafer texturing and p-n junction fabrication

In this work, we incorporate the BNNN processing into the standard c-Si solar cell fabrication. We begin this fabrication with silicon substrate texturing. A silicon Si (100) wafer (p-type, 1 Ω·cm resistance, 15.6 × 15.6 cm^2^) was textured in a standard alkali solution, and then before the PECVD doping to form the p-n junction, its native oxide was etched away in a 0.5 % diluted HF. The texturing resulted in micro-pyramid formation (typically ~1.5 μm tall and ~3 μm wide at base). PCl_3_ gas was used in the PECVD chamber as the n-doping source and the junction formed at the depth of ~300 nm. The processed wafer was subsequently washed in 1 % diluted HF solution to remove the phosphorous silicate glass (PSG), the unwanted by-product of the doping process. This was followed by the immersion of the wafer in de-ionized water for 10 min to remove the residual HF.

### Nanoparticle deposition and sintering

In the next step, the silver nanoparticle ink was spray-deposited on the surface of the textured and doped wafer. Within a few minutes, the capillary forces cause the nanoparticles to agglomerate in the deep valleys in between the texture pyramids. This process is enhanced with mechanical shaking of the wafer. At sufficient density, NP form continuous chains. However, a simple touching is not sufficient to assure good inter-particle contact, since NPs are coated with PVP insulating shells (by-product of their synthesis). To remove these, and to form continuous metallic wires by pre-melting, we implemented anneal/sintering in a microwave oven and in a furnace. The microwave sintering [29, 30] was done in a commercial microwave oven operating at 2.46 GHz, with the output power of 80 W. A typical exposure time used was ~10 s to selectively heat and sinter the silver nanoparticles into a continuous conducting nanowire network. The furnace sintering was done in a commercial rapid thermal processor (RTP) (RTP-500, Beijing Eaststar Labs) at temperature of 320 °C and duration of 10 s. The sintering resulted in the formation of well-conducting NNN.

### Metal-assisted chemical etching

In the next step, we employed the metal-assisted chemical etching (MacEtch method [25, 26]) to “bury” the NNN under the silicon surface. The textured Si sample with NNN was immersed into an ethanol-based etchant composed of 4.6 M HF and 0.1 M H_2_O_2_. The chemical or electrochemical reactions occur preferentially near the noble metal NP in an etchant consisting of HF and H_2_O_2_. The possible cathode and anode reactions are as follows [25, 26]: H_2_O_2_ + 2H^+^ → 2H_2_O + 2 h^+^ at the metal (cathode reaction), and at the anode, the Si substrate is directly oxidized and dissolved in a tetravalent state as follows Si + 4 h^+^+4HF → SiF_4_ + 4H^+^ and SiF_4_ + 2HF → H_2_SiF_6_. With the Si oxidized and dissolved at the Si/metal interface by HF, the NNN sinks down (becomes “buried”) into the silicon substrate. Simultaneously, NNN scale-sized pits form on the silicon surface (as shown in Fig. 1f). The pit depth increases gradually with the duration of etching. To minimize the energy consumption, and to make the process more compatible with a large scale manufacturing, we chose the processing temperature to be ~25 °C (room temperature). The etching lasted from 1 to 60 s. Then, the sample was washed in distilled water several times to remove etching solution, followed by etching in 5 % HF for 2 min to remove the silicon oxide layer. This was followed by a repeated washing in distilled water and finally drying at 150 °C for 2 min. This processing resulted in BNNN. The etching time rates can be varied with the concentration of etchant.

### SiN processing

In the next standard c-Si processing step, the hydrogen-doped SiN was thermally deposited and processed. This high-quality dielectric film serves double function. Firstly, it forms an ARC, which reduces the light reflection, and secondly, the hydrogen released from SiN during the heat processing passivates the silicon-nitride interface imperfections and thus increases dramatically the carrier lifetime. In our structure, modified by the presence of BNNN, the curing process occurs around the BNNN. This is different than in the standard process, where no metal is present at the silicon-nitride interface until the silver paste micro-particles deposited on the nitride are subsequently thermally forced (“fired”) to diffuse through the nitride. Both these processes (silver paste screen printing and “firing”) have been eliminated in our processing.

A continuous square Al film is used as a back contact deposited by thermal evaporation method. A crossed Ag strips (2 mm × 1.5 cm) is used as the front contacts to the BNNN on the front also by thermal evaporation. Before Ag strips deposition, the underneath SiN layer is etching away with protection of a crossed hollow mask.

### Performance measurements

The morphologies of samples were characterized by a commercial SEM system (JEOL JCM-5700, Tokyo, Japan). We used an X-ray diffraction system (PANanalytical, X`Pert-Pro MPD PW 3040/60 XRD with Cu-Kα1 radiation, the Netherlands) to do material analysis. The dark and illuminated *I*-*V* measurements of the solar cells were done with the solar simulator (Oriel Newport, USA). Both evaporations were done in thermal vacuum evaporations system (SKY Vacuum Technology Company, China). The reflectance was measured by employing the fiber optic spectrometer (Ocean Optics, USB 4000) and the integration sphere (Ocean Optics, FOIS-1). Silver NP area coverage was calculated by analyzing the SEM images via Photoshop CS5 software.

## Results and discussion

### Sample morphologies

**Fig. 2:**
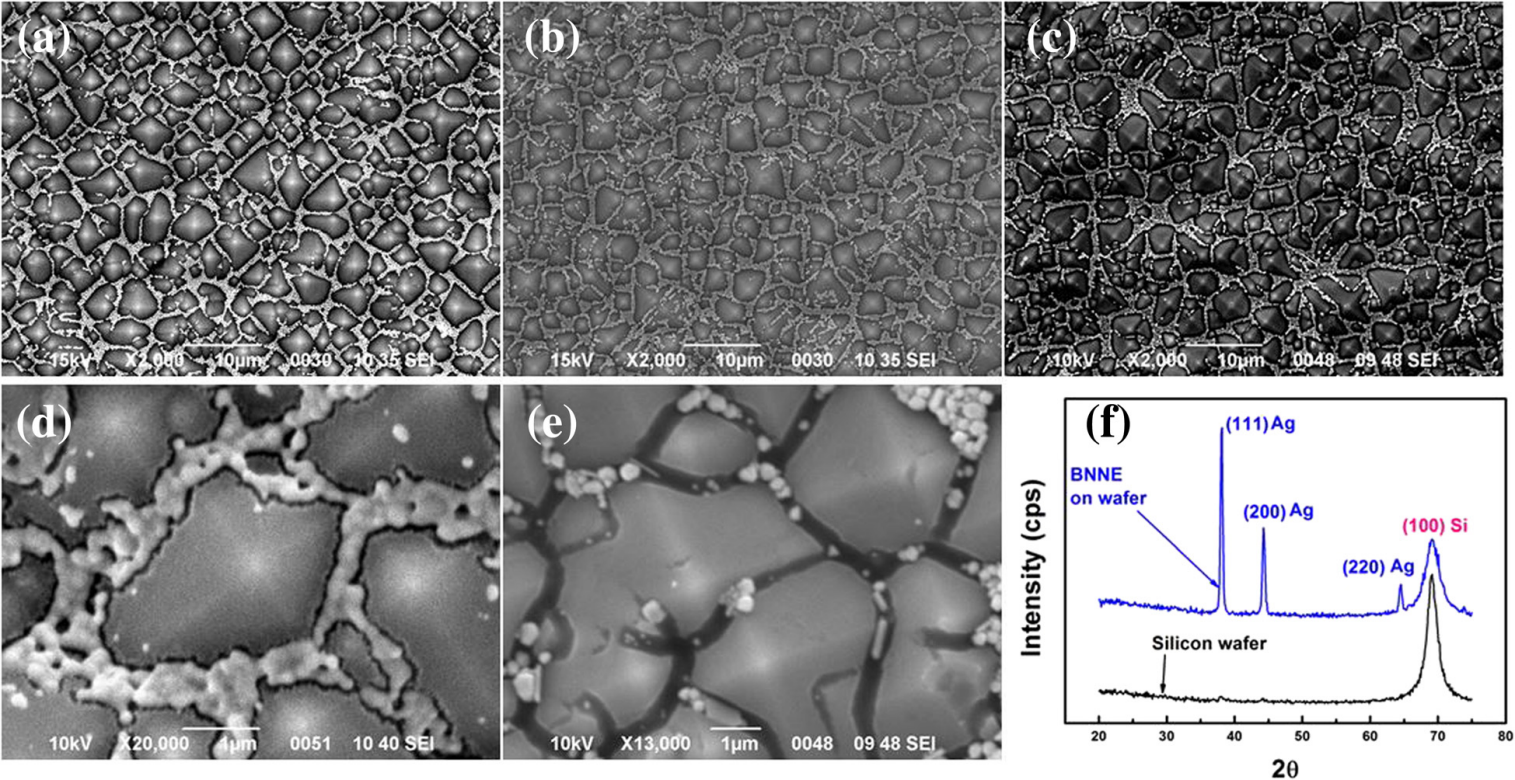
SEM images of various morphologies of Ag nanoparticles on tSi at different stages of the processing: **a** chains of nanoparticles self-assembled in the inter-pyramid valleys, **b** NNN after sintering by RTP, **c** BNNN, **d** large magnification image of BNNN, **e** large magnification image of BNNN after removal of NP, showing the etching pits, and **f** XRD pattern of BNNN on tSi as compared to that for the bare silicon wafer. The scale bars in **a**–**c** are 10 μm and in **d** and **e** are 1 μm

Figure 2 shows SEM images of nanoparticle morphologies on the textured silicon (tSi) after various steps of processing. Figure 2a shows the formation of continuous paths of touching nanoparticle chains in the inter-pyramid valleys (SEM images of the enlarged nanoparticle cluster around a pyramid are shown in Additional file 1: Figure S1a and b). Such a connected network forms when NP density exceeds the percolation threshold. Figure 2b shows NNN after sintering, and the corresponding BNNN is shown in Fig. 2c. Figure 2d is a magnified image of the BNNN, showing well-sintered (pre-melted) NP. Figure 2e shows also a magnified image of the BNNN, but with most of the NP etched away, showing clearly the etched pits (The cross-sectional BNNN sample is shown in Additional file 1: Figure S1c, and the red arrow points the buried silver nanoparticle cluster). The X-ray diffraction patterns were taken from BNNN on tSi and also from a bare Si are shown in Fig. 2f. The peaks can be assigned to (111), (200), and (220) planes of silver and the (100) plane of silicon. The absence of the Ag_2_O and SiO_2_ peaks demonstrates that neither the nanowires nor silicon have been oxidized during the sintering and the metal-assisted etching process. In addition, the XRD patterns also demonstrate that our samples contain only silver and silicon elements and are free of undesired impurities.

### Optical performance

**Fig. 3:**
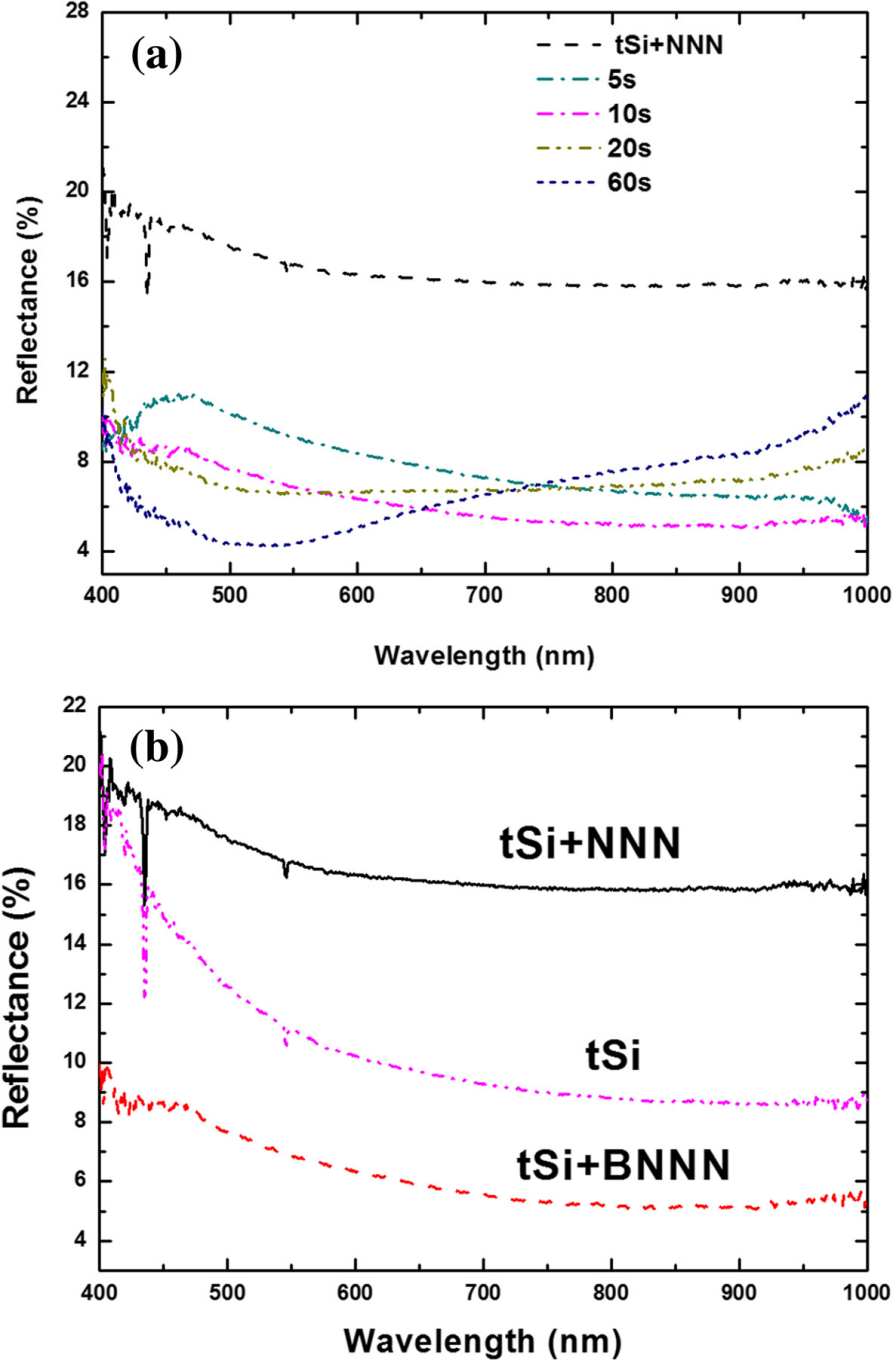
Reflectance spectra of **a** BNNN on tSi with p-n junction as a function of etching time and **b** NNN, BNNN, and bare tSi for etching time chosen to be 10 s

To demonstrate the excellent optical performance of BNNN structures, we used in a series of samples the tSi substrate obtained by interrupting the standard solar cell processing (as described above) just before the SiN deposition. As discussed above, we expect that reflectance from such BNNN will be strongly reduced. Indeed, this effect is demonstrated in Fig. 3a, which shows evolution of the reflectance spectra of the BNNN, as a function of etching time; the reflectance for the etching times over 10 s time drops to less than 10 %. Note that in this test, we have not measured the conductivity of the BNNN TCE. This excellent optical performance of our BNNN is primarily due to the buried/subsurface nature of the metallic network (a direct reduction of the scattering), as well as the nanowires placement deep in the valleys between pyramids of the texture. This location causes a significant part of the reflected/scattered light from the network to be reabsorbed by the pyramids, thus enhancing the overall transmission into the silicon substrate. In addition, a plasmonic action similar to that involved in the extraordinary optical transmission further improves transmission into silicon [31]. The reduction of the reflectance is only in small part due to increased absorption of radiation in silver. This has demonstrated experimentally in our previous work, by directly measuring the transmission through the silicon substrate in the near-infrared frequency range [24]. Figure 3b compares the reflectance spectra of NNN, BNNN, and bare tSi. The reflectance of BNNN on tSi surface is clearly lower than that of NNN on tSi, but amazingly also much lower than that of the bare tSi.

### Electrical performance

**Fig. 4:**
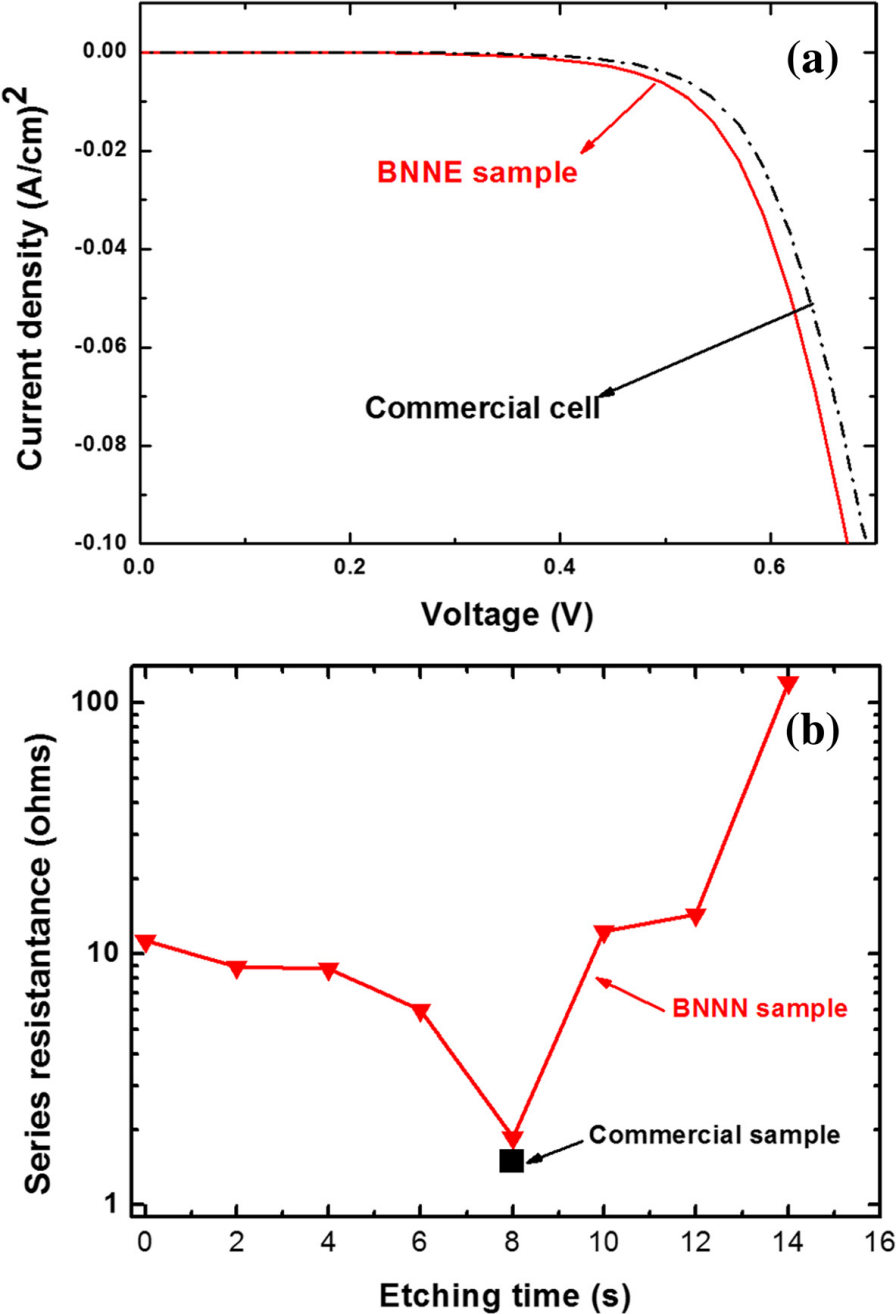
**a** Dark *I*-*V* characteristics of the commercial (*dashed-dotted line*) and our best BNNN structure (*solid line*, *t*_e_ = 8 s, optimal for *R*_s_). **b** *R*_s_ of BNNN structure (*solid line*) as a function of *t*_e_, as well as that of a standard commercial cell (*solid square*)

The same samples were used to demonstrate an excellent electrical performance of BNNN, in this case used as the top contact to the p-n junction (with Al contact on the back). The current voltage (*I*-*V*) characteristics in the absence of illumination (dark) have been measured for various BNNN etching times. Figure 4a shows the best dark *I*-*V* characteristic (8-s etching time), compared with the dark *I*-*V* of a standard commercial cell. Clearly, the characteristics are almost identical, and in particular, the series resistance (*R*_s_ = dV/dI, in the rapidly changing current section of the curve) is practically the same as that of the commercial cell (~1.8 Ω). Figure 4b shows *R*_s_ as a function of the etching time (*t*_e_). Clearly, *R*_s_ is not a monotonic function of the increasing *t*_e_, with a sharp minimum (~1.8 Ω) at *t*_e_ = 8 s, essentially as low as for the commercial cell (solid square). This behavior is easy to understand as follows. As the BNNN “sinks” into silicon during the etching, *R*_s_ first diminishes, as the network approaches the junction located ~300 nm below the surface, and then rapidly increases when the network crosses, and thus damages the junction. While at the etching time of 8 sec *R*_s_ is minimal, the reflectance after SiN deposition, while not minimal. is still low, about 12 %. This shows that BNNN can be an excellent TCE for c-Si solar cells.

The initial, fast decrease of *R*_s_ (shown in Fig. 4b) and the slight shift of the dark *I*-*V* characteristics of the BNNN cell towards lower voltages, as compared with that for a conventionally processed cell, indicate a large carrier recombination. This is an expected result of the commercial cell surface texturing. This recombination rate can be dramatically reduced by a passivation treatment with SiN [32, 33].

### Performance of the BNNN solar cells

**Fig. 5:**
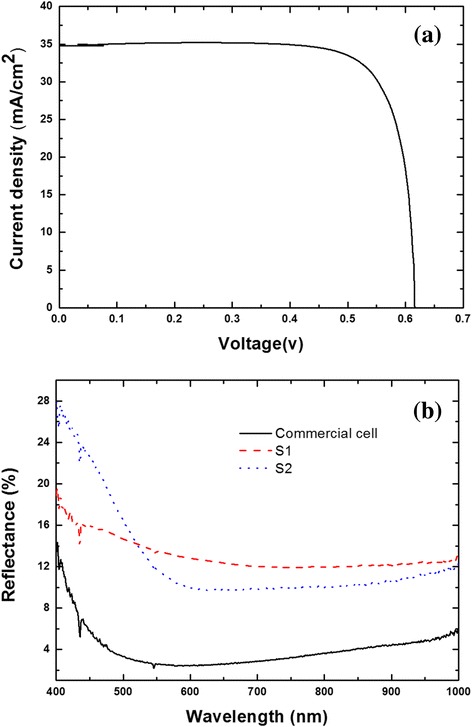
**a** *I*-*V* characteristics of the conventionally processed (on a commercial c-Si production line) control solar cell at “one sun” illumination (AM1.5). **b** Reflectance of BNNN solar cells (with etching time of 8 s, *dashed line* and *dotted line*) and the control (*solid line*)

After demonstrating the excellent electro-optical performance of BNNN on tSi, we have fabricated complete BNNN solar cells, using a commercial c-Si processing line. This was done by interrupting the conventional cell processing just before the nitride processing (like in the electro-optical performance tests) and deposited BNNN at optimal condition *t*_e_ = 8 s. Note that the optimal condition is for *R*_s_, not for reflectance. Subsequently, the wafer was returned back to the solar cell processing, commercial line for SiN deposition, and then removed again for reflectance measurements, contact deposition, and finally efficiency measurements. The conventionally processed, full wafer-sized control cell has the *I*-*V* characteristic (at “one sun” illumination, AM1.5) shown in Fig. 5a, and the efficiency of 17.85 %, which is similar to the standard commercial cells. Since our current BNNN manufacturing set-up assures high uniformity only in regions of size of about 1 cm × 2 cm, this sets up an upper limit on the size of our BNNN cells, which have been cut out of the processed wafers. In order to achieve the size parity with the conventionally processed, control cell, we have cut it also to the same size 1 cm × 2 cm as chosen for the BNNN cells and have processed contacts in the same way. Now, the cell efficiencies, measured on a small solar simulator, can be characterized by the efficiency reduction factor *r* = *η*/*η*_control_, where *η* is the BNNN cell efficiency. Table 1 displays values of *r* for the best BNNN cells S1 as S2. The *I*-*V* curves of a typical BNNN and control cells with sample size of about 1 cm × 2 cm are shown in Additional file 1: Figure S2.

**Table 1:** Efficiency reduction factors for BNNN cells

Solar cell type	Efficiency reduction factor *r* = *η*/*η*_control_
c-Si conventional	1
c-Si BNNN (S2)	0.86
c-Si BNNN (S1)	0.72

Lower efficiency of the BNNN cells is primarily due to lower current density, which is clearly due to the higher reflectance of these cells, shown in Fig. 5. Note, that the sample “tSi + BNNN”, reflectance of which is shown in Fig. 3b, is different from samples S1 and S2 (Fig.5b). The sample “tSi + BNNN” is obtained with etching time of 10 s, not optimal for minimal *R*_s_, but samples S1 and S2 were obtained with etching time of 8 s, optimal for *R*_s_.

As discussed above, there is a rather narrow window where the etching time *t*_e_ yields BNNN which is both very conductive and weakly reflecting. In the electro-optical performance tests, this time was *t*_e_ = 8 s, but in the samples for the solar cell tests, which went through additional step of SiN processing, was most likely different. According to Fig. 4b, this dramatically changes the electro-optical performance. We expect that with better understanding and control of the BNNN processing, cell efficiencies can be increased to those of the conventionally processed cells, or possibly higher, keeping in mind the excellent result shown in Fig. 3b.

We stress that our BNNN solar cells are fabricated using a solution-processed method, less expensive than the standard production line process. We have estimated the cost of the full size BNNN solar cell to be ~$0.4, with ~$0.17 cost of nanoparticles and the rest the standard commercial processing costs, minus the screen printing and firing expenses. Cost of the conventionally processed solar cell (same size), including the screen printing and firing expenses is ~$0.5. Thus, the BNNN solar cell processing costs are about 80 % of those of the conventionally processed cell, a potential dramatic advantage once the BNNN process is perfected to be used on full wafers, and with the BNNN cell efficiencies at least that of the conventional (commercial) solar cells.

## Conclusions

In conclusion, we have demonstrated a novel, highly transparent and conducting electrode (BNNN), which provides a high-quality, ohmic contact to a textured crystalline silicon substrate. We demonstrate also preliminary results on the c-Si solar cells with BNNN, obtained by replacing the usual commercial screen-printed macro-electrode with our BNNN. The efficiency of our BNNN cell is only 14 % lower than that for the conventional/commercial control, while the manufacturing cost is only ~80 % of the commercial, because it avoids the two most expensive steps of the conventional processing: screen printing and subsequent firing. With better understanding and improvements in BNNN processing, we expect efficiencies of these cells to reach, or possibly exceed, the commercial cell efficiencies.

## Abbreviations

ARC, anti-reflection coating; BNNN, buried nanoparticle nanonetwork; CNT, carbon nanotubes; c-Si, crystalline silicon; ITO, indium tin oxide; NNN, nanoparticle nanonetwork; NP, nanoparticles; PSG, phosphorous silicate glass; PV, photovoltaics; PVP, polyvinylpyrrolidone; RTP, rapid thermal processor; TCE, transparent conductive electrodes; tSi, textured silicon

## Additional file

Additional file 1: The file contains supplementary Figures S1–S2. (DOCX 256 kb)
