# Supplementary material for: Inexpensive transparent nanoelectrode for crystalline silicon solar cells
Source: Nanoscale Res Lett. 2016 Jun 29;11:312. doi: 10.1186/s11671-016-1533-3 (PMC4927558; doi:10.1186/s11671-016-1533-3)
Supplement: Additional file 1: — The file contains supplementary Figures S1–S2. (DOCX 256 kb) [file 11671_2016_1533_MOESM1_ESM.docx]

Additional file 1:

**Inexpensive Transparent Nano-electrode for Crystalline Silicon Solar Cells**

Qiang Peng, ^1^ Ke Pei, ^1, 5^ Bing Han, ^1^ Ruopeng Li, ^1^ Guofu Zhou, ^2^ Jun-Ming Liu, ^1, 4^ Krzysztof Kempa, ^3, 1^ and Jinwei Gao^* 1^

^1^ Institute for Advanced Materials (IAM) and Laboratory of Quantum Engineering and Quantum Materials, South China Normal University, Guangzhou 510006, P. R. China

^2^ Electronic Paper Displays Institute, South China Normal University, Guangzhou 510006, P. R. China

^3^ Department of Physics, Boston College, Chestnut Hill, MA 02467, USA

^4^ Laboratory of Solid State Microstructures, Nanjing University, Nanjing 210093, P. R. China

^5^ Laboratory of Nanoscale Energy Conversion Devices and Physics, Department of

Mechanical Engineering, The University of Hong Kong, Pokfulam, Hong Kong

Email addresses for all authors:

QiangPeng: pengqiang33714@163.com

Ke Pei: 523907144@qq.com

Bing Han: zhihan.kong1894@qq.com

Ruopeng Li: li_ruopeng@163.com

Guofu Zhou: zhougf@scnu.edu.cn

Jun-Ming Liu:[liujm@nju.edu.cn](mailto:liujm@nju.edu.cn)

Krzysztof Kempa: kris.kempa@bc.edu

JinweiGao: [gaojw@scnu.edu.cn](mailto:gaojw@scnu.edu.cn)

The author of JinweiGaowas the corresponding author.


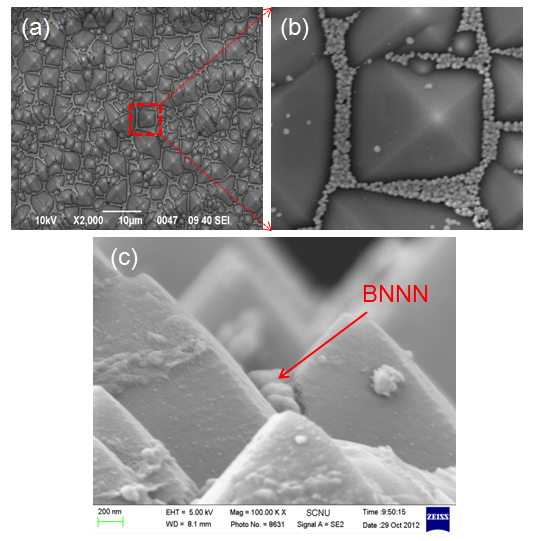


Figure S1 SEM images of (a) large-area BNNN sample, (b) a selected area enlarged BNNN sample and (c) a cross sectional BNNN sample. The red arrow points the buried silver nanoparticle cluster.

**
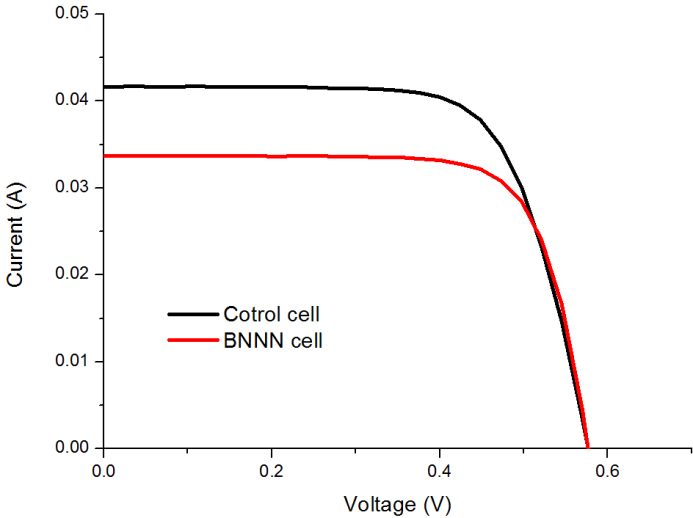
**

Figure S2 The IV curves of control cell and BNNN cell.

The control samples were made by cutting the conventional solar cell into pieces of identical size, and electrode configuration as the BNNN cells.
